# Assessing risk of liver enzyme elevation in patients with immune-mediated diseases and different hepatitis B virus serostatus receiving anti-TNF agents: a nested case-control study

**DOI:** 10.1186/s13075-017-1413-y

**Published:** 2017-11-01

**Authors:** Ying-Ming Chiu, Mei-Shu Lai, K. Arnold Chan

**Affiliations:** 1 Division of Allergy, Immunology and Rheumatology, Changhua Christian Hospital, 135 Nanxiao St, Changhua, 500-06 Taiwan; 2 Department of Nursing, College of Medicine and Nursing, Hungkuang University, Taichung, Taiwan; 3 Graduate Institute of Epidemiology and Preventive Medicine, College of Public Health, National Taiwan University, Taipei, Taiwan; 4 Department of Medical Research, National Taiwan University Hospital, Taipei, Taiwan; 5 Graduate Institute of Oncology, College of Medicine, National Taiwan University, Taipei, Taiwan

**Keywords:** Hepatitis B virus, HBsAg^+^, HBsAg^–^/HBcAb^+^, Liver enzyme elevation, Anti-TNF

## Abstract

**Background:**

Liver enzyme elevation is an important and common adverse effect among patients with immune-mediated diseases who receive tumour necrosis factor inhibitors (anti-TNF), and has various causes. Hence, we evaluated the relative risks of developing liver enzyme elevation in anti-TNF users with differing hepatitis B virus (HBV) infection status.

**Methods:**

At a hospital in central Taiwan, 407 patients with rheumatoid arthritis, ankylosing spondylitis, or psoriasis/psoriatic arthritis received anti-TNF therapy between 1 January 2004 and 30 June 2012. We performed a nested case-control study (*n* = 368) of cases with serum alanine aminotransferase (ALT) > 40 international units/L ≤ 12 months after starting anti-TNF therapy, and corresponding controls without liver enzyme elevation. Conditional logistic regression was used to evaluate associations between liver enzyme elevation and HBV serostatus, as well as other risk factors.

**Results:**

Thirty cases were compared to 338 controls. After adjustment for potential confounders, HBV surface antigen-positive (HBsAg^+^) serostatus was associated with substantially higher likelihood of developing elevated ALT (adjusted odds ratio 7.91, 95% confidence interval (CI) 2.16–31.31) relative to those with an uninfected HBV status; no such association was observed among HBsAg-negative/HBV core antibody-positive (HBsAg^–^/HBcAb^+^) patients (adjusted odds ratio 1.00, 95% CI 0.33–3.25). Increased risk of ALT elevation was associated with methotrexate used alone, without folic acid (adjusted odds ratio 11.60, 95% CI 2.52–56.46), and history of ALT elevation (adjusted odds ratio 13.71, 95% CI 4.32–45.75).

**Conclusions:**

HBsAg^+^ patients with immune-mediated diseases who received anti-TNF therapy had an approximately eight-fold higher likelihood of liver enzyme elevation than those without HBV infection, whereas patients with HBsAg^–^/HBcAb^+^ serostatus had a risk similar to that of uninfected patients.

**Electronic supplementary material:**

The online version of this article (doi:10.1186/s13075-017-1413-y) contains supplementary material, which is available to authorized users.

## Background

Tumour necrosis factor inhibitors (anti-TNF), including infliximab, etanercept, adalimumab, and golimumab, are widely used to treat immune-mediated diseases, including rheumatoid arthritis (RA), ankylosing spondylitis (AS), psoriasis (PsO), and psoriatic arthritis (PsA). Cases of liver enzyme elevation have been reported after anti-TNF use, and possible causes include reactivated hepatitis B virus (HBV) infection [1], idiosyncratic hepatotoxicity related to concomitant immunosuppressive agents (for example, methotrexate (MTX) [2]), chronic liver diseases such as fatty liver or alcoholic liver disease [3], and autoimmune hepatitis [4].

Patients with chronic HBV infection—i.e. HBV surface antigen seropositive (HBsAg^+^)—are at risk of HBV reactivation consequent to immunosuppression, and should therefore be given prophylactic antiviral treatment before receiving conventional or biologic immunosuppressant therapies [5]. However, the absolute risk of liver damage in patients with differing HBV infection status is uncertain. Although heightened risk of hepatitis among HBsAg^+^ patients receiving anti-TNF agents has been reported in individual cases and small clinic-based cohorts, the risk estimates provided by such studies are imprecise [1, 6–11]; for example, the risk may be influenced by sex, age, type of immune-mediated disease and, especially, concomitant hepatotoxic agents [12]. No published studies have investigated the correlation between HBsAg^+^ status and liver enzyme elevation after controlling for the aforementioned factors. Although HBV surface antigen-negative/HBV core antibody-positive (HBsAg^–^/HBcAb^+^) patients may also be at risk, the risk appears to be relatively low with inconsistent findings. In particular, some investigators have reported cases of hepatitis due to HBV reactivation [1, 13], whereas others report no cases [7, 14]. Concomitant immunosuppressive therapy, especially with hepatotoxic agents, is associated with the risk of hepatitis consequent to HBV reactivation in HBsAg^–^/HBcAb^+^ patients [15].

Without appropriately controlling for potential risk factors, it is impossible to compare the risk of developing liver enzyme elevation in patients with differing HBV infection status receiving anti-TNF therapy. This clinical question is important in Taiwan, where HBV infection is hyperendemic; up to 15–20% of the population is HBsAg^+^ and seroprevalence of HBcAb before universal HBV vaccination commenced in 1984 was 80–90% [16–18]. We identified a large cohort of hospital patients and conducted a nested case-control study to evaluate the associations between differing HBV serostatus and risk of alanine aminotransferase (ALT) elevation in patients receiving anti-TNF therapy.

## Methods

### Patient identification and study design

We retrospectively reviewed medical records from Changhua Christian Hospital, which is a major medical centre in central Taiwan, and identified patients treated since 1999 for RA, AS, and PsO/PsA, all of whom fulfilled international diagnostic criteria for these conditions. A retrospective anti-TNF cohort comprising 407 patients who were first treated with etanercept, adalimumab, or golimumab from 1 January 2004 (when anti-TNF therapy for RA was first approved in Taiwan) through to 30 June 2012 was followed-up until 30 June 2013 (12 months). Before starting anti-TNF therapy, all of these patients had received conventional disease-modifying anti-rheumatic drugs (DMARDs), including MTX, sulfasalazine (SSZ), leflunomide (LEF), cyclosporine (CYS), hydroxychloroquine (HCQ), or cyclophosphamide, and most continued DMARD treatment concomitantly with anti-TNF.

**Fig. 1:**
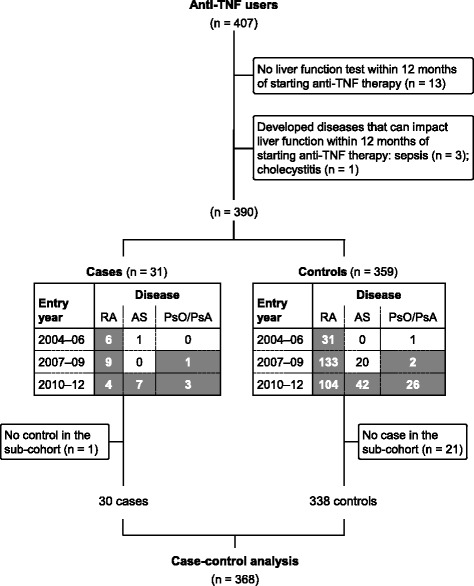
Cohort identification and ascertainment of cases and controls. *Bold white* numbers indicate selected case and control patients, *plain* numbers indicate cases with no control in the sub-cohort and vice versa. *AS* ankylosing spondylitis, *PsO* psoriasis, *PsA* psoriatic arthritis, *RA* rheumatoid arthritis, *TNF* tumour necrosis factor

The study cohort was restricted to patients who had been followed-up for at least 12 months since commencing anti-TNF therapy, including those with continued follow-up after anti-TNF agents were withdrawn due to ALT elevation within 12 months of starting treatment. As Taiwan National Health Insurance requires all patients who receive reimbursed anti-TNF drugs to provide blood samples 6 months later and 3-monthly thereafter, most have routine liver enzyme assays during the first year; patients with no such test during this period were excluded, as were those who developed diseases which could lead to liver enzyme elevation within 12 months after starting anti-TNF treatment (Fig. 1).

### Nested case-control design

Due to the complexity and varying durations of drug exposures in this cohort, we applied a new-user design with nested case-control analysis, which affords equivalent validity to a cohort analysis without compromising statistical power [19, 20]. Patients were stratified into nine sub-cohorts (Fig. 1) based on disease type (RA, AS, and PsO/PsA) and calendar year of first anti-TNF use (2004–2006, 2007–2009, and 2010–2012).

### Case and control definitions and ascertainment

Abnormal liver enzyme elevation was defined as serum ALT exceeding twice the upper limit of normal (ULN)—i.e. > 40 international units/L—within 1 year of starting anti-TNF treatment, as per another study of hepatotoxicity associated with anti-TNF therapy in RA [21]; the first date when serum ALT was observed to exceed twice the ULN was designated the “event date”. This timeframe was chosen because HBV-related liver enzyme elevation mostly arises within the first few months of anti-TNF therapy [22].

From each of the nine sub-cohorts of patients, those who developed ALT elevation within 1 year after starting anti-TNF treatment were cases, and subjects from the same subgroup who did not were controls. For each control, a random date within 12 months after starting anti-TNF therapy was selected and designated the index date.

### Exposure measurement

Based on serology analyses by chemiluminescent microparticle immunoassay (Architect i2000SR, Abbott Laboratories, Abbot Park, Illinois, USA) that were carried out before anti-TNF therapy began, patients were divided into three HBV infection status categories: 1) HBsAg^+^ and HBcAb^+^, denoted HBsAg^+^; 2) HBcAb^+^ but HBsAg^–^, denoted HBsAg^–^/HBcAb^+^; or 3) both HBsAg^–^ and HBcAb^–^, denoted uninfected.

### Covariate information

Potential confounders that were evaluated included sex, age, history of ALT elevation (serum ALT at least twice the ULN within 12 months before starting anti-TNF therapy), and use of the immunosuppressant drugs MTX, prednisolone (PRED), HCQ, SSZ, LEF, CYS, and azathioprine (AZA). Three different categories of MTX use were defined: 1) no MTX; 2) MTX concurrent with folic acid; and 3) MTX alone without folic acid; other immunosuppressant drugs were defined as either used or not used. Use of MTX and PRED (continuous variables) was investigated, including accumulated doses for the past 6 months, and long-term doses accumulated since the earliest record for each patient [23, 24]. Use of non-biologic immunosuppressants was defined as recorded treatment within 30 days before the event date (cases) or the index date (controls) [25–27].

### Statistical analysis

Conditional logistic regression was used to estimate the odds ratios (OR) and 95% confidence intervals (CIs) for occurrence of ALT elevation in patients with differing HBV serostatus. In Model 1, crude ORs associated with HBsAg^+^ and HBsAg^–^/HBcAb^+^ were estimated, with uninfected status as the reference. In Model 2, we estimated adjusted ORs by introducing potential individual confounders (sex, age, medical history of ALT elevation, PRED, MTX, HCQ, SSZ, LEF, CYS, AZA) along with HBV infection status in bivariate analyses to identify significant confounders. Model 3 was a multivariate analysis that included sex, age, and selected confounders based on bivariate analyses in Model 2. Due to the sparse data in some sub-cohorts, all statistical analyses were conducted using nonparametric statistics software (LogXact; Version 10.1, Cytel Software Corp, Cambridge, MA, USA) with penalised maximum likelihood to remove first-order bias. The sub-cohort was treated as a stratum variable. In all analyses, *p* < 0.05 for two-sided tests was considered statistically significant.

## Results

### Demographic characteristics and clinical data of study subjects

Table 1 summarises the demographic and clinical characteristics of the 368 subjects with immune-mediated diseases (RA, AS, PsO/PsA) who were included in the case-control analysis. Females and patients with RA predominated in both groups. Around half of cases and controls had started anti-TNF therapy after 2010. Compared with the controls, the cases included proportionally more HBsAg^+^ patients, fewer HBsAg^–^/HBcAb^+^ patients, and more patients with a medical history of ALT elevation; additionally they were younger on average. Drug use was generally similar between cases and controls; however, proportionally more cases than controls used MTX alone without folic acid, whereas relatively more controls than cases were prescribed concurrent MTX and folate. Also, proportionally more cases than controls received AZA and fewer received HCQ.

**Table 1:** Baseline characteristics of case and control subjects

	Cases (*n* = 30)	Controls (*n* = 338)	*p* value
HBV infection status			<0.001
HBsAg^+^	8 (22.7%)	21 (6.2%)	
HBsAg^–^/HBcAb^+^	14 (46.7%)	210 (62.1%)	
Uninfected	8 (26.7%)	107 (31.7%)	
Sex			0.617
Female	20 (66.7%)	240 (71.0%)	
Male	10 (33.3%)	98 (29.0%)	
Age (years), mean ± SD	46.6 ± 15.9	52.2 ± 15.2	0.001
Immune-mediated disease			0.011
Rheumatoid arthritis	19 (63.3%)	268 (79.3%)	
Ankylosing spondylitis	7 (23.3%)	42 (12.4%)	
Psoriasis/psoriatic arthritis	4 (13.3%)	28 (8.3%)	
Year anti-TNF therapy began			0.038
2004–2006	6 (20.0%)	31 (9.2%)	
2007–2009	10 (33.3%)	135 (39.9%)	
2010–2012	14 (46.7%)	172 (50.9%)	
Medical history of elevated ALT	8 (26.7%)	18 (5.3%)	<0.001
Immunosuppressant drug use			
Prednisolone	21 (70.0%)	246 (72.8%)	0.744
Methotrexate	21 (70.0%)	243 (71.9%)	0.825
Methotrexate alone, without folic acid	9 (30.0%)	28 (8.3%)	<0.001
Methotrexate + folic acid	12 (40.0%)	215 (63.6%)	
Hydroxychloroquine	13 (43.3%)	209 (61.8%)	0.047
Sulfasalazine	18 (60.0%)	222 (65.7%)	0.531
Cyclosporine	4 (13.3%)	55 (16.3%)	0.674
Leflunomide	2 (6.7%)	30 (8.9%)	0.681
Azathioprine	1 (3.3%)	1 (0.3%)	0.030
Immunosuppressant drug dose received (mg), mean ± SD			
Methotrexate dose			
6-month accumulated	197.7 ± 148.8	210.0 ± 149.2	0.665
Total accumulated	1160.7 ± 1263.3	1445.5 ± 1390.7	0.280
Prednisolone dose			
6-month accumulated	837.8 ± 551.1	830.9 ± 679.5	0.957
Total accumulated	6679.5 ± 6510.5	7482.9 ± 7879.2	0.588

Values are shown as *n* (%) unless otherwise stated

*ALT* alanine aminotransferase, *HBcAb* ^*+*^ HBV core antibody positive, *HBsAg* ^*+*^ */* ^*–*^ HBV surface antigen positive/negative, *HBV* hepatitis B virus, *SD* standard deviation, *TNF* tumour necrosis factor

During the 12-month follow-up period, the 30 cases had 131 liver enzyme assays and the 338 controls had 1469 (approximately 4.3 per patient on average). No HBsAg^+^ patients received antiviral prophylaxis during the first 12 months of anti-TNF therapy; however, many did receive such prophylaxis subsequent to publication of the Taiwan Rheumatology Association guidelines in 2012 [28]. Additional file 1 (Table S1) summarises the clinical status of the 30 cases before, during, and after they developed ALT elevations. The majority had ALT elevations ≥ 2.5 × ULN, eight with ALT > 5 × ULN; however, no cases of liver enzyme elevation had fatal outcomes and ALT levels in most patients normalised either spontaneously or after moderating the treatment regimen. Only four of eight HBsAg^+^ cases were tested for virology; three had detectable HBV DNA, and two received antiviral therapy because of HBV reactivation (HBV DNA > 100,000 copies/ml).

### The association between HBV infection status and liver enzyme elevation in patients receiving anti-TNF therapy

The crude ORs for different HBV infection statuses and ALT elevation were estimated by conditional logistic regression (Table 2). Univariate analysis showed HBsAg^+^ status to be a significant risk factor for ALT elevation; however, there was no significant correlation between ALT elevation and HBsAg^–^/HBcAb^+^ status. In bivariate analysis that included individual potential confounders to HBV infection status in the regression models, HBsAg^+^ remained significantly associated with ALT elevation regardless of which additional variables were controlled for. Significant confounders associated with the risk of ALT elevation in bivariate models were use of MTX only (OR 6.95, 95% CI 1.70–29.65) and history of elevated ALT (OR 9.66, 95% CI 3.24–28.83).

**Table 2:** Univariate and bivariate analyses of HBV infection status and ALT elevation

Analysis model (*n* = 368)		HBsAg^+^ vs uninfected	HBsAg^–^/HBcAb^+^ vs uninfected
	OR (95% CI)	OR (95% CI)	OR (95% CI)
Model 1 (univariate analysis): HBV infection status		5.05 (1.68–15.42)	0.97 (0.40–2.51)
Model 2 (bivariate analysis): HBV infection status adjusted for each confounder:			
Sex (female vs male)	1.21 (0.48–3.26)	5.03 (1.68–15.28)	0.96 (0.40–2.48)
Age (10-year intervals)	0.81 (0.59–1.11)	6.41 (1.99–21.57)	1.29 (0.47–3.77)
History of elevated ALT^a^	9.66 (3.24–28.83)	4.83 (1.54–15.37)	0.79 (0.30–2.11)
Prednisolone	1.62 (0.62–4.58)	5.29 (1.75–16.22)	0.97 (0.40–2.48)
Methotrexate^a^		4.86 (1.58–15.06)	0.77 (0.30–2.04)
Methotrexate alone	6.95 (1.70–29.65)		
Methotrexate + folic acid	1.17 (0.38–3.82)		
Hydroxychloroquine	0.79 (0.30–2.21)	4.95 (1.65–15.06)	0.97 (0.40–2.51)
Sulfasalazine	0.66 (0.30–1.48	5.48 (1.79–17.14)	1.02 (0.42–2.65)
Cyclosporine	1.05 (0.30–3.05)	5.02 (1.67–15.29)	0.97 (0.40–2.51)
Leflunomide	0.75 (0.13–2.82)	5.13 (1.71–15.59)	0.98 (0.40–2.50)
Azathioprine	0.13 (0.01–1.78)	5.43 (1.80-16.65)	1.02 (0.42–2.62)
Methotrexate 6-month accumulated dose	1.21 (0.89–1.68)	5.57 (1.82–17.38)	0.98 (0.41–2.53)
Methotrexate total accumulated dose	0.99 (0.96–1.03)	4.97 (1.65–15.15)	0.98 (0.40–2.53)
Prednisolone 6-month accumulated dose	1.15 (0.64–1.96)	5.15 (1.70–15.83)	0.98 (0.40–2.52)
Prednisolone total accumulated dose	1.00 (0.94–1.05)	5.00 (1.67–15.20)	0.97 (0.40–2.51)

^a^ Confounding factors with statistical significance

*ALT* alanine aminotransferase, *CI* confidence interval, *HBcAb* ^*+*^ HBV core antibody positive, *HBsAg* ^+^/^*–*^ HBV surface antigen positive/negative, *HBV* hepatitis B virus, *OR* odds ratio

The multivariate model included HBV infection status, age, sex; two significant confounding factors were found to be associated with ALT elevation in the bivariate models (history of elevated ALT during the 12 months before anti-TNF therapy started, and MTX use). HBsAg^+^ serostatus was significantly associated with the risk for developing ALT elevation, with an OR of 7.91 (95% CI 2.16–31.31), whereas the adjusted OR for HBsAg^–^/HBcAb^+^ versus uninfected was 1.00 (Table 3). Moreover, the risk associated with MTX use alone without folic acid was substantially increased but the risk associated with MTX concomitant with folic acid was not elevated. Furthermore, compared with no history of elevated ALT, prior history was a significant risk factor for developing ALT elevation during anti-TNF treatment. In addition, risk of ALT elevation declined significantly with advancing age.

**Table 3:** Multivariate analysis of HBV infection status and liver enzyme elevation (Model 3)

Patient characteristics (*n* = 368)		OR (95% CI)
HBV infection status	HBsAg^+^ vs uninfected	7.91 (2.16–31.31)
	HBsAg^–^/HBcAb^+^ vs uninfected	1.00 (0.33–3.25)
Sex	Female vs male	0.91 (0.34–2.54)
Age^a^	20-year intervals	0.47 (0.24–0.91)
History of elevated ALT	History vs no history	13.71 (4.32–45.75)
Methotrexate	Methotrexate + folic acid vs no methotrexate	2.00 (0.61–7.20)
	Methotrexate alone without folic acid vs no methotrexate	11.60 (2.52–56.46)

^a^ Age categorised by 20-year intervals; 1 = 0–20 years, 2 = 20–40 years, 3 = 40–60 years, 4 = 60–80 years, 5 = ≥ 80 years

*ALT* alanine aminotransferase, *CI* confidence interval, *HBcAb* ^+^ HBV core antibody positive, *HBsAg* ^+^/^–^ HBV surface antigen positive/negative, *HBV* hepatitis B virus, *OR* odds ratio

## Discussion

This study, which was conducted in an HBV endemic region, is the first to have determined the relative risk of liver enzyme elevation in large cohorts of anti-TNF users with differing HBV serostatus, controlling for potential risk factors. The influence of HBV infection status on such a risk has never been estimated accurately; the results of published studies may have been influenced by concomitant hepatotoxic pharmacotherapy, underlying disease, and other patient characteristics. Moreover, this study reflects the precise risk of ALT elevation in HBV-infected patients because most were prescribed anti-TNF agents before formal risk management guidelines were issued in Taiwan and, therefore, received no prior antiviral prophylaxis [28]. HBsAg^+^ anti-TNF users had an almost eight-fold higher likelihood of ALT elevation than uninfected patients. MTX use without supplementary folic acid, and previous history of elevated ALT, were associated with a more than ten-fold higher risk of ALT elevation.

In previous studies, the major concern when HBsAg^+^ anti-TNF users developed liver enzyme elevation was HBV reactivation; however, the rates reported in these small studies differed considerably. In two studies conducted in Taiwan [1, 10], HBV reactivation incidence rates were 62.5% (*n* = 5/8) and 50% (*n* = 3/6), respectively; however, both studies investigated patients with only one immune-mediated disease (RA or PsO/PsA). Two Korean studies in AS patients reported relatively lower HBV reactivation rates of 6.9% (*n* = 2/29) and 12.5% (*n* = 1/8), respectively [6, 11]. Therefore, different types of immune-mediated diseases appear to influence the likelihood of HBV reactivation. Patient demographics differ between diseases [1, 6, 11]; for instance, most RA patients are female, whereas male AS patients predominate. Patients with different diseases are also likely to have proportionally different exposure to hepatotoxic immunosuppressant drugs—MTX is often given to RA and PsO/PsA patients but less often to AS patients. Moreover, previous studies showed that each type of immune-mediated disease had a different incidence of hepatotoxicity, despite treatment with the same immunosuppressant drugs [29]. Furthermore, it is likely that studies from different countries used different treatment strategies and doses of hepatotoxic immunosuppressants [30–32]. Therefore, analysis of the risk of hepatitis in HBsAg^+^ subjects must take into account many potential risk factors, including sex, age, their underlying immune-mediated disease, and concomitant use of hepatotoxic drugs. For these reasons, we controlled for such potential risk factors when evaluating the risk of liver enzyme elevation. Since no cases or controls received antiviral prophylaxis before the event date or index date, the role of antiviral prophylaxis was not evaluated.

In contrast with HBsAg^+^ status, the HBsAg^–^/HBcAb^+^ serotype was not associated with increased likelihood of ALT elevation in patients receiving anti-TNF therapy. A recent meta-analysis of nine studies that involved 486 HBsAg^–^/HBcAb^+^ subjects who received anti-TNF therapies reported that HBV reactivation incidence was only 1.7% (*n* = 8/468) [15]. Given such a low incidence of HBV reactivation and the moderate sample size of our current study, it would be difficult to detect a significant association between HBsAg^–^/HBcAb^+^ and risk of ALT elevation. Furthermore, we used serum ALT as an indicator which will underestimate the incidence of HBV reactivation in HBsAg^–^/HBcAb^+^ subjects because this is defined by the increase in the viral DNA level or conversion from HBsAg^–^ to HBsAg^+^; thus ALT levels may remain normal during HBV reactivation [13, 33].

As in prior studies, we found that MTX use without folic acid was positively associated with the risk of ALT elevation [34]; however, no study has compared the risk of MTX-related hepatitis in anti-TNF users with differing HBV infection status. Although we found that MTX use was associated with a higher risk of ALT elevation than HBsAg^+^ status, previous reports suggested that cases of MTX-related hepatitis were mostly mild [35]. Besides, our observation that ALT levels normalised after withdrawing MTX in most cases may indicate that ALT elevations were due more to MTX hepatoxicity than other factors; this may explain why our findings differ from those of a recent Japanese study that reported a lower risk of HBV reactivation with concomitant MTX and a higher risk with prednisolone [36]. However, as we did not directly evaluate HBV reactivation, patients in either study probably had different risk factors.

We also found that a history of elevated ALT was a risk factor for ALT elevation in patients receiving anti-TNF agents, as was also shown by another study in new MTX users [34]. This finding suggests that ALT elevations may be due to other patient-related risk factors, including excessive alcohol consumption, unhealthy lifestyle, or diseases such as fatty liver disease or cirrhosis [3]. Risk of ALT elevation declined with advancing age, which is consistent with other reports that ALT levels correlate negatively with age [37]; epidemiologic studies of non-alcoholic fatty liver disease have consistently shown an inverse association between age and steatosis [38–40].

These study findings should be considered in light of notable limitations, principally arising from the retrospective design, which makes it difficult to draw firm conclusions; in particular, we accrued only 30 cases (22 HBV seropositive) and several clinical data were incomplete. First and foremost, ALT elevation does not necessarily reflect HBV reactivation, and lack of viral load data in most cases with elevated ALT is a major limitation to interpreting its clinical significance; for example, chronic liver cirrhosis is another possible cause of ALT elevation in HBsAg^+^ versus uninfected subjects. Second, we have no data on other common causes of ALT elevation such as obesity or alcohol intake, although a history of elevated ALT in some patients (Additional file 1: Table S1) might be due to such underlying causes. Third, although we investigated the effects of HBV serostatus and immunosuppressants as risk factors, we did not specifically evaluate the risk of immunosuppressant use in HBsAg^+^ patients, which is known to increase the risk of HBV reactivation [5]. Fourth, although viral load and HBsAb status may influence the risk of HBV reactivation in HBsAg^–^/HBcAb^+^ patients, we lack viral load data for these patients, and had HBsAb results for only 10/224, among whom nine, including three cases, were positive, and one negative. Fifth, although patients had four liver enzyme assessments on average within 12 months after anti-TNF therapy commenced, 13/407 patients had no test (Fig. 1). Lastly, infliximab, which is reported to have the highest risk of HBV reactivation, is not available in Taiwan and so was not included.

## Conclusion

This hospital-based nested case-control study demonstrates that HBsAg^+^ patients with immune-mediated diseases receiving anti-TNF therapies are approximately eight times more likely than uninfected patients to develop ALT elevation, whereas those with HBsAg^–^/HBcAb^+^ serostatus have similar risk to that of uninfected patients. MTX monotherapy without folic acid and a history of elevated ALT also correlate with ALT elevation.

## Additional file

Additional file 1: Table S1. Clinical status of 30 patients who developed abnormal liver function during treatment with anti-TNF agents. Table S1 summarises relevant clinical data on 30 cases before, during, and after developing liver enzyme elevation. (PDF 632 kb)

## Abbreviations

ALT: Alanine aminotransferase
Anti-TNF: Tumour necrosis factor inhibitor
AS: Ankylosing spondylitis
AZA: Azathioprine
CI: Confidence interval
CYS: Cyclosporine
DMARD: Disease-modifying anti-rheumatic drug
HBV: Hepatitis B virus
HBcAb^+^: HBV core antibody positive
HBsAg^+^: HBV surface antigen positive
HBsAg^−^: HBV surface antigen negative
HCQ: Hydroxychloroquine
LEF: Leflunomide
MTX: Methotrexate
OR: Odds ratio
PRED: Prednisolone
PsA: Psoriatic arthritis
PsO: Psoriasis
RA: Rheumatoid arthritis
SSZ: Sulfasalazine
ULN: Upper limit of normal
