# Supplementary material for: Assessing risk of liver enzyme elevation in patients with immune-mediated diseases and different hepatitis B virus serostatus receiving anti-TNF agents: a nested case-control study
Source: Arthritis Res Ther. 2017 Nov 1;19:214. doi: 10.1186/s13075-017-1413-y (PMC5664916; doi:10.1186/s13075-017-1413-y)
Supplement: Additional file 1: Table S1. — Clinical status of 30 patients who developed abnormal liver function during treatment with anti-TNF agents. Table S1 summarises relevant clinical data on 30 cases before, during, and after developing liver enzyme elevation. (PDF 632 kb) [file 13075_2017_1413_MOESM1_ESM.pdf]

Supplementary Table S1. Clinical status of 30 patients who developed abnormal liver function during treatment with anti-TNF agents

Disease	HBV serostatus			Prior ALT elevation[a]	Hepatic profile when ALT elevation occurred					Medication profile when ALT elevation occurred						Management response to ALT elevation	ALT level after management response
					AST/ALT (x ULN)	Total bilirubin (mg/dl)	Coagulation	Virology	Liver sonography[b]	Anti-TNF		MTX		PRED dose (mg/dy)	Other DMARD(s)		
	HBsAg	HBcAb	HBsAb							agent	duration (mo)	dose (mg/wk)	with folate				
RA	+	+	ND	No	NR/2.6	NR	NR	ND	PLD	ETA	7.0	7.5	Yes	5	SSZ	Stop ETA/MTX	Normal
AS	+	+	ND	No	3.5/5.5	0.47	NR	HBV DNA: 6491 IU/ml<br>HBeAg−, HBeAb+	Normal	ADA	3.7	2.5	Yes	0	SSZ	Stop ADA/MTX	Normal
RA	+	+	ND	Yes	1.7/2.3	NR	NR	ND	PLD	ETA	0.7	7.5	Yes	7.5	HCQ/SSZ	Stop MTX, start antiviral therapy	Normal
RA	+	+	ND	No	NR/2.0	NR	NR	ND	Normal	ETA	1.6	7.5	No	0	None	Stop ETA	Normal
RA	+	+	ND	No	NR/2.3	NR	NR	ND	ND	ETA	0.9	7.5	No	5	SSZ	None	Normal
RA	+	+	ND	No	2.2/3.7	NR	NR	HBeAg−	Normal	ETA	11.2	0	NR	5	LEF/HCQ/SSZ	Stop ETA/PRED/LEF	Normal
RA	+	+	ND	No	22.6/33.9	1.68	PT: 11.4<br>INR: 1.2	HBV DNA: 186940 IU/ml<br>HBeAg+	Fatty liver	ETA	4.7	10	Yes	2.5	HCQ/SSZ	Stop ETA/MTX, start antiviral therapy	Normal
RA	+	+	ND	Yes	6.8/8.5	8.3	PT: 14.3<br>INR: 1.3	HBV DNA: 5522132 IU/ml<br>HBeAg−	Normal	ETA	0.9	15	Yes	10	HCQ/SSZ	Stop MTX/ETA, start antiviral therapy	Normal
AS	−	+	ND	No	NR/2.8	NR	NR	ND	Fatty liver	ADA	1.9	15	Yes	5	SSZ	Stop MTX	Normal
AS	−	+	ND	No	NR/2.4	NR	NR	ND	ND	ADA	10.3	0	NR	0	None	None	Normal
RA	−	+	ND	No	NR/2.5	NR	NR	ND	ND	ADA	12.0	15	Yes	5	HCQ	None	Normal
PsO	−	+	ND	No	2.6/2.8	NR	NR	ND	PLD	ADA	1.9	10	No	0	SSZ	MTX 10→7.5 mg	Normal
RA	−	+	ND	No	NR/3.2	NR	NR	HBV DNA: negative	PLD	ADA	7.5	15	No	7.5	None	MTX 15→10 mg	Normal
PsO	−	+	+	Yes	3.1/3.0	NR	NR	ND	PLD	ADA	7.5	0	NR	0	CYS	None	Normal
RA	−	+	ND	Yes	NR/2.1	NR	NR	ND	Fatty liver	ETA	1.6	7.5	No	10	SSZ	Stop SSZ, add folate	Normal
RA	−	+	+	Yes	NR/9.0	NR	NR	ND	Normal	ETA	6.3	10	No	5	HCQ/SSZ	Stop MTX	Normal

Chiu YM, Lai MS, Chan KA. Assessing risk of liver enzyme elevation in patients with immune-mediated diseases and different hepatitis B virus serostatus receiving anti-TNF agents: a nested case-control study. DOI: 10.1186/s13075-017-1413-y

RA	−	+	ND	No	NR/5.5	NR	NR	ND	PLD	ETA	1.9	10	No	7.5	HCQ/SSZ	MTX 10→5 mg	Normal
RA	−	+	ND	No	NR/2.2	NR	NR	ND	ND	ETA	8.4	10	Yes	5	SSZ	Stop ETA, MTX 10→7.5 mg	Normal
RA	−	+	ND	No	NR/3.6	NR	NR	ND	Fatty liver	ETA	10.3	10	Yes	5	HCQ	MTX 10→5 mg	Normal
RA	−	+	ND	Yes	1.6/2.1	NR	NR	ND	ND	ETA	6.5	10	Yes	0	HCQ/SSZ	MTX 10→5 mg	Normal
PsA	−	+	ND	Yes	2.9/5.7	NR	NR	HBV DNA: negative	Normal	ETA	0.7	0	NR	5	HCQ/SSZ/CYS	Stop ETA	Normal
AS	−	+	+	No	NR/5.5	NR	NR	ND	Fatty liver	ETA	4.4	0	NR	0	SSZ	Stop ETA	Normal
PsA	−	−	ND	No	NR/3.0	NR	NR	NR	ND	ADA	11.7	0	NR	5	CYS/LEF	None	Normal
RA	−	−	ND	No	1.3/2.5	NR	NR	NR	Fatty liver	ADA	2.8	10	No	5	HCQ, SSZ	MTX 10→5 mg	Normal
RA	−	−	ND	No	NR2.5	NR	NR	NR	ND	ETA	4.7	15	Yes	5	None	MTX 15→12.5 mg	Normal
JRA	−	−	ND	No	NR/3.6	NR	NR	NR	Normal	ETA	7.6	10	No	5	AZA/HCQ	None	Normal
JRA	−	−	ND	No	NR/2.2	NR	NR	NR	ND	ETA	3.7	5	Yes	1.25	None	None	Normal
AS	−	−	ND	No	NR/2.0	NR	NR	NR	ND	ETA	4.7	0	NR	0	SSZ	SSZ 1 g→500 mg	Normal
AS	−	−	ND	No	NR/7.1	NR	NR	NR	ND	ETA	4.4	0	NR	0	SSZ	SSZ 2 g→1 g	Normal
PsO	−	−	+	Yes	1.5/3.2	NR	NR	NR	ND	ETA	11.2	0	NR	0	None	None	Abnormality persisted

TNF, tumor necrosis factor; HBV, hepatitis B virus; HBsAg, HBV surface antigen; HBcAb, HBV core antibody; HBsAb, HBV surface antibody; ALT/AST, alanine/aspartate aminotransferase; ULN, upper limit of normal; mo, month; wk, week; dy, day; DMARDs, disease-modifying anti-rheumatic drugs; MTX, methotrexate; PRED, prednisolone; HCQ, hydroxychloroquine; SSZ, sulfasalazine; CYS, cyclosporine; AZA, azathioprine; LEF, leflunomide; ETA, etanercept; ADA, adalimumab; HBeAg, HBV e antigen; HBeAb, HBV e antibody; RA, rheumatoid arthritis AS, ankylosing spondylitis; PsO, psoriatic arthritis; JRA, juvenile rheumatoid arthritis; PLD, parenchymal liver disease[b]; PT, prothrombin time; INR, international normalized ratio; IU, international units; ND, not done; NR, not reported.

[a] ALT >two-fold ULN during the past year of anti-TNF treatment.

[b] In Taiwan, ultrasound findings intermediate between “normal” and “cirrhosis” based on sonographic evaluation criteria for liver surface, liver parenchyma, hepatic vessels and spleen size, are diagnosed as “parenchymal liver disease”. These criteria are described in detail in: Hung CH, Lu SN, Wang JH, et al. Correlation between ultrasonographic and pathologic diagnoses of hepatitis B and C virus-related cirrhosis. J Gastroenterol. 2003;38:153–7.

Chiu YM, Lai MS, Chan KA. Assessing risk of liver enzyme elevation in patients with immune-mediated diseases and different hepatitis B virus serostatus receiving anti-TNF agents: a nested case-control study. DOI: 10.1186/s13075-017-1413-y
